# Early Female Transgender Identity after Prenatal Exposure to Diethylstilbestrol: Report from a French National Diethylstilbestrol (DES) Cohort

**DOI:** 10.3390/jox14010010

**Published:** 2024-01-12

**Authors:** Laura Gaspari, Marie-Odile Soyer-Gobillard, Scott Kerlin, Françoise Paris, Charles Sultan

**Affiliations:** 1 Unité d’Endocrinologie-Gynécologie Pédiatrique, CHU Montpellier, University Montpellier, 34090 Montpellier, France; laura.gasparisultan@gmail.com (L.G.); f-paris@chu-montpellier.fr (F.P.); 2 INSERM 1203, Développement Embryonnaire Fertilité Environnement, University of Montpellier, 34295 Montpellier, France; 3 CHU Montpellier, University Montpellier, Centre de Référence Maladies Rares du Développement Génital, Constitutif Sud, Hôpital Lapeyronie, 34295 Montpellier, France; 4 Laboratoire Arago, Observatoire Océanologique, Sorbonne University, CNRS, 75016 Paris, France; elido66@orange.fr; 5 Association HHORAGES-France, 66100 Perpignan, France; 6 DES International Information and Research Network, Livermore, CA 94551, USA; skerlin@gmail.com

**Keywords:** sexual identity, diethylstilbestrol (DES), prenatal exposure

## Abstract

Diagnostic of transsexualism and gender incongruence are terms to describe individuals whose self-identity does not match their sex assignment at birth. A transgender woman is an individual assigned male at birth (AMAB) on the basis of the external or internal genitalia who identifies and lives as a woman. In recent decades, a significant increase in the number of transgender people has been reported. Although, its etiology is unknown, biological, anatomical, genetic, environmental and cultural factors have been suggested to contribute to gender variation. In XY animals, it has been shown that environmental endocrine disruptors, through their anti-androgenic activity, induce a female identity. In this work, we described four XY individuals who were exposed in utero to the xenoestrogen diethylstilbesterol (DES) and were part of the French HHORAGES cohort. They all reported a female transgender identity starting from childhood and adolescence. This high prevalence of male to female transgenderism (1.58%) in our cohort of 253 DES sons suggests that exposure to chemicals with xenoestrogen activity during fetal life may affect the male sex identity and behavior.

## 1. Introduction

Gender identity defines each individual’s deeply held personal sense of their own gender as male or female or something else [1]. Moreover, gender diversity and variance are umbrella terms used to describe the wide range of gender identifications outside the conventional gender categories [2]. Gender dysphoria [3] relates to the distress and unease experienced by individuals who are discontent with their assigned gender and identify with a gender other than the one associated with their birth sex [4]. A transgender man or woman is a person born phenotypically female or male, assigned female or male at birth on the basis of their external or internal genitalia, who identifies and lives as a male or a female. Actually, for many experts [5] this definition should be enlarged to Transgender and Gender Non-Conforming (TGNC) individuals, people with disorders of sex development (DSDs) and people assigned male (AMAB) or female at birth (AFAB) [6].

All studies on secular trends have reported a dramatic increase in the number of transgender people in recent decades [7]. A temporal change in the age of presentation is another notable phenomenon [8]. Quoting Hassler et al. [8] in the *Diagnostic and Statistical Manual of Mental Disorders*, Fifth Edition, Text Revision (DSM-5), signs of transgender in children include: “A repeated desire to be the other sex or an assertion that they are the other sex; a belief that their assigned sex will change on its own (e.g., thinking they will grow a penis or that their penis will come off when they get older); wanting to change their name to a name typically associated with another gender, or a gender-neutral name; a preference for presenting as another gender (e.g., a child assigned male at birth who prefers long hair and wearing dresses, or a child assigned female at birth who prefers short hair and wearing gender-neutral clothing); resistance or distress when made to present as a gender they do not identify with (such as a ‘girl’ throwing a tantrum over having to wear a dress or a ‘boy’ crying after having to get a haircut); assuming the role of another gender in fantasy games or make-believe; an intense desire to participate in the games and activities typical of the other gender (such as an assigned-male child playing with Barbie and an assigned-female child playing contact sports); a preference for playmates of another gender”.

Hassler et al. also wrote [8]: “In adolescence, signs of transgender includes: feelings of panic or severe discomfort concerning puberty and body changes (e.g., refusing to acknowledge or admit that changes are occurring, refusing to look at their body, or becoming distressed or uncomfortable with body development, menstruation, or ejaculation); discomfort or distress with certain forms of gender presentation, such as disliking long hair or certain types of clothing; increased bullying at school due to differences in acting out one’s perceived gender or lack of self-confidence; isolation from peers due to lack of connection or fear of bullying; depression and/or anxiety as a result of confusion over gender identity or not fitting in with peers; be aware that some adolescents may try to repress their true gender due to outside pressure, such as from family and peers, even if they previously expressed their true gender as a child”.

The literature on the prevalence of transgender people is heterogeneous, depending on the geographic area, inclusion criteria and age at presentation [9]. Specialized centers that manage transgender people estimate a prevalence between 17 and 33/100,000 individuals [10]. Actually, people who identify as transgender represent a sizable proportion of the general population, from 0.1% to 2% [11,12].

Although the biological basis of transgenderism/incongruence is unknown, biological, anatomical, genetic and environmental factors have been suggested to contribute to gender identity [13,14]. The role of sex hormones and genetics in sexual development was described by studies published between 1948 and 2019, and the implication of endogenous steroids in brain sexual differentiation has been widely studied [14,15,16,17,18]. According to the accepted dogma, high levels of fetal testosterone organize the brain towards a male phenotype [19]. Any exogenous chemical that can reduce testosterone action during fetal life can affect the differentiation of genitalia and sex behavior.

Diethylstilbestrol (DES) is a molecule that has strengthened the concepts of endocrine-disrupting chemicals (EDCs) and the fetal basis of adult diseases [20]. It is well known that in utero exposure to DES, a compound with estrogenic and anti-androgenic activity, induces a wide range of reproductive tract/function abnormalities in the so-called ‘DES daughters’, e.g., alterations to Müllerian duct development, fertility problems, ectopic pregnancies, miscarriages, premature births and cancers, especially clear cell adenocarcinoma (CCA) of the vagina and cervix in girls and young women [21]. In ‘DES sons’, epididymal cysts, hypospadias, cryptorchidism, hypoplastic testes and micropenises have been observed [22,23,24,25,26]. In addition, although less studied, in utero exposure to synthetic sex hormones, particularly DES, can cause psychological disorders, such as schizophrenia, bipolar disorders, eating disorders and suicidal behavior [27]. Interestingly, some studies in patients exposed in utero to DES with psychotic disorders identified methylome changes that affect the expression of ZFP57 and ADAMTS9, two genes implicated in neurodevelopment regulation [28], with potential multigenerational and transgenerational effects [29,30].

In this work, we first describe four transgender women identified among the 253 sons exposed in utero to DES included in the HHORAGES-France cohort (a French National patient association). This high prevalence of male-to-female transgender individuals among DES sons (1.58%) strongly suggests that exposure of male fetuses to this xenoestrogen during fetal life may affect future male sex identity and behavior.

## 2. Patients and Methods

This study was based on a French national retrospective cohort of DES-treated women (HHORAGES-France Association) (n = 1200) and their families. Detailed standard questionnaires were obtained from all DES mothers when they joined the HHORAGES-France Association and all DES children were already adults at this time. The answers to this online questionnaire were used to select women who met the following inclusion criteria: (1) at least two pregnancies with two viable male babies (same father) among whom the first child was not exposed in utero to DES (pre-DES), followed by one or more children with fetal exposure to DES (DES); (2) confirmed data on the total DES dose (health record or physician’s observation). The cumulative dose D administered to the French pregnant mothers was 4050 mg < D < 7300 mg. This is considered a medium-low-dose cohort, in accordance with the observations by Tournaire et al. [31] and compared with the higher doses administered in the United States (7550 mg < D < 12,742 mg). The answers to the questionnaires were also used to identify women with a DES son who presented female gender variance. We then contacted all DES sons with female gender variance and we carried out an individual interview with them.

The local university hospital ethics committee approved this study (ID IRB No. 202301531), and all patients gave their informed consent through the HHORAGES-France Association (CNIL: J B/EM/DC042793, N° 1006460).

All DES sons who identified as transgender women underwent karyotyping at their local hospital at the moment of transition.

### 2.1. Patient 1 (M.) (Table 1, First Column)

Currently a writer and photographer, M. was born in 1961 after in utero exposure to DES given to their mother following a previous miscarriage. At birth, M. had male genitalia and unilateral cryptorchidism. At the age of 4 years, M. started to present gender dysphoria that became clear at the age of 9 years (Table 1, first column). During adolescence, M. expressed the conviction of being a woman. This period was also characterized by important school absenteeism and poor academic performance. In adult life, M. presented severe psychological disorders, particularly a severe self-mutilation episode (external genitalia) at the age of 43 years (2004), with a major, life-threatening hemorrhage. After seeing a psychiatrist, M. began the transition with male to female gender reassignment surgery in France, after the agreement of the French court of first instance. Since this operation, she has been receiving Gender-Affirming Hormone Therapy (GAHT) and she is regularly followed as part of GAHT management.

### 2.2. Patient 2 (S.) (Table 1, Second Column)

Currently a composer (guitar and vocals), S. was born in 1969 after in utero exposure to DES. At birth, S. had male genitalia with unilateral cryptorchidism. S. started to question the assigned male gender at the age of 4 years: “I remember very clearly that when I was 3–4 years old, one day I went with my mother to a hairdressing salon and having looked at all the ladies, I thought: ‘When I grow up this is what I will do: I will be a woman’”.

During adolescence, S. felt he was a woman and had severe psychological disorders, particularly depression and suicidal ideation (Table 1, second column). As an adult, S. married and had two children. These two girls had prolactinoma, and one has Asperger’s syndrome, androgyny and ovarian cysts. S. began the transition with male to female gender reassignment surgery, in Brighton, United Kingdom (UK), in November 2015. Since then, S. has been receiving GAHT and is followed by the doctor who managed the transition. According to the UK Gender Recognition Act, S. could change their sex recorded on their birth certificate (male to female) and now, she lives in Scotland. Her elder sister, also exposed to DES in utero after her mother’s miscarriage, died due to vaginal adenocarcinoma during adolescence.

### 2.3. Patient 3 (Chr.) (Table 1, Third Column)

Currently a ULM pilot teacher, Chr. was born in 1963 after in utero exposure to DES and slow-release progestin. At birth, Chr. had male genitalia without any genital malformation and spina bifida. Chr. reported gender dysphoria at the age of 7 years (see Table 1, third column). During adolescence, Chr. had psychological disorders and enuresis up to the age of 16 years. At the age of 22 years, Chr. married and had one son at the age of 23 years. From the age of 30 years, Chr. regularly dressed as a female and finally divorced after 20 years of married life. Chr. began the transition with male to female gender reassignment surgery performed in Thailand, in 2003, at the age of 40 years. Since this operation, she has been receiving GAHT. She lives in a couple with her former female partner and the French court of first instance of her department recognized her identity change on June 2006, at the age of 43 years.

### 2.4. Patient 4 (J.) (Table 1, Fourth Column)

J. was born in May 1953 in the USA (Kansas, Mid-West) after in utero exposure to DES. At birth, J. had normal male genitalia and no malformation. Gender dysphoria began at about 3–4 years of age. During childhood, J. always liked to wear his sister’s clothing, including panties, slips and dresses (Table 1, fourth column). J. had no girlfriend at the age of 16 years, and later, fantasized to be Annette Funicello, a very feminine movie star. During adolescence, J. always fantasized by looking at women’s apparel and dreamt of wearing all the items featured in catalogues, especially panties and lingerie, and often dressed as a woman. Once an adult, J. married, “But still society pushed me into marriage which is where I am now, taking cross-sex hormones to become a girl!”. Indeed, from 1997, J. has been taking estradiol to change sex, but did not undergo gender reassignment surgery.

## 3. Results

**Figure 1:**
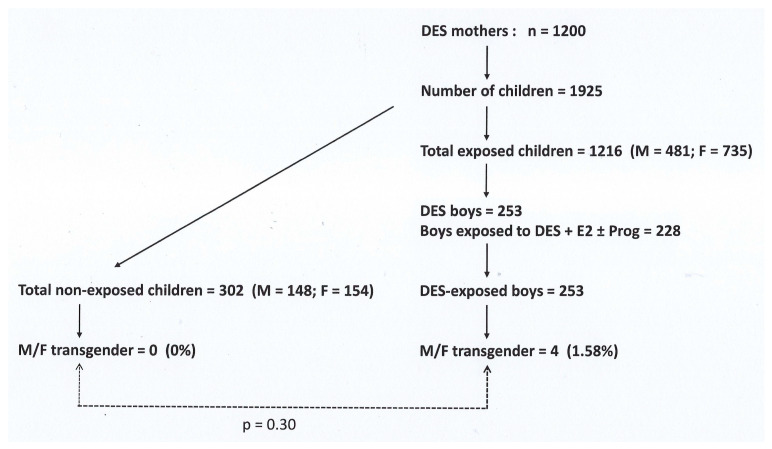
Included and excluded patients of the HHORAGES-France cohort in which four transgender M/Fs were identified. (HHORAGES-France data.) E2 is the synthetic hormone 17-α-estradiol that was often administered in a cocktail with DES and synthetic progesterone during pregnancy, but was banned for pregnant women in Europe in 1980.

In this French national retrospective cohort of DES-treated women (i.e., DES mothers; n = 1200) and their families, 253 boys who were exposed in utero to DES (DES sons) and 148 pre-DES sons were identified. The flowchart showing the included and excluded patients is shown in Figure 1. Four of these DES sons identified themselves as transgender women (transgender prevalence =1.58%) versus zero among the pre-DES sons (0%). The prevalence difference was not significant (*p*-value = 0.30), probably because of the small sample size. The DES doses were documented for all 253 DES sons.

In these four DES sons who identified themselves as transgender women, karyotyping did not reveal any abnormality. Major transgender components and behaviors were present already in early childhood and adolescence, as described in the previous section and summarized in Table 1.

## 4. Discussion

The number of patients seeking gender-specific healthcare has drastically increased (30–40-fold) in the last three decades [32]. In a recent European study, 0.7–1% of the general population reported incongruent gender identity [33]. However, several reports highlighted the important heterogeneity in the published estimates across studies. Moreover, the definition of transgender is often expanded to include broader manifestations of gender diversity. However, non-binary people should not be included in this wide definition. If we consider the highest prevalence of transgender women reported in the literature (1/17,000) [10], the prevalence we observed in our study (1.58%) is 10- to 100-fold higher. Moreover, the prevalence of female transgender identity was 0% among the 148 elder non-exposed sons (AMAB) in the same informative families.

It is generally accepted that fetal androgens play a crucial role in male brain differentiation by directly activating neuronal regions to provide organizational changes. In male individuals, gender identity, gender behavior and sex orientation are driven by prenatal androgen action [34]. Basic gender identity is developed at the age of 2–3 years [35]. By this age, most children have become aware of gender stereotypes, for instance, through toy preference and clothing. It is interesting to note that in our four patients, most diagnostic signs of transgenderism were present early in life and persisted during adolescence and adult life. In addition, mental health disorders, particularly depression, anxiety and suicidal ideation, have frequently been reported by DES children [27,36] and also by our four patients, starting from early adolescence and then in adulthood. Moreover, in many countries, transgender people live at the margin of society, and despite a higher educational level, they have a lower socio-economic status.

Although research in the transgender field is increasing, the causal mechanisms have not been adequately identified yet [13,37]. Genetic [38,39], biological [1,4], anatomical [40,41] and environmental factors [42,43] have mainly been studied. A greater chance of transgender concordance among XY monozygotic tweens than dizygotic tweens [44] supports a genetic component. A significant association between female transgenderism and over-represented alleles and genotypes (estrogen receptor α, ERα; androgen receptor, AR; Steroid 5 Alpha-Reductase 2, SRD5A2; cytochrome 17, CYP17) has been identified by Foreman et al. [38]. These authors suggested that gender dysphoria may have a polygenetic basis that may alter the masculinization of the fetal brain, contributing to the development of a female transgender identity. Moreover, the second-to-fourth digit length ratio (2D:4D), a proxy of prenatal androgen activity, was recently found to be higher, i.e., feminized, in female transgender persons. This finding reinforces the role of reduced prenatal androgen activity in female transgenderism. Unfortunately, we could not scan the left hand of our patients [45]. The primacy of testosterone [19] activity during fetal life is a critical driver of brain androgenization and the subsequent male sex identity and behavior [39,40].

In many animal studies, EDC exposure during development has been associated with changes in sex behavior, such as disruption of the normal social preference behavior (rats) and alteration of the differentiation of relevant sexually dimorphic pathways (mouse, Japanese quail) [42,43]. The most convincing evidence linking testosterone to the establishment of the male gender identity comes from individuals with DSDs, such as XY complete androgen insensitivity syndrome [45], XY 5 alpha reductase deficiency [46] and XX congenital adrenal hyperplasia (CAH) [47]. For example, individuals with 5 alpha reductase deficiency are usually reared as girls on the basis of their female external genitalia [47]. They change the initial gender assignment during puberty, when testosterone concentration increase can organize the brain structures and influence the adolescent gender identity and behavior. XX fetuses with CAH and exposed to a high testosterone level during prenatal life present a high risk of gender incongruence. The transgender prevalence among individuals with DSDs is much higher than in the general population, although there are substantial differences among DSD etiologies. In a European collaborative work from the DSD Life Study, 4% of the 1040 participants with DSDs reported gender variance [48]. In our four patients, no DSD was diagnosed at birth. Patients 1 and 2 presented unilateral cryptorchidism, which cannot be considered a clinical expression of a DSD on its own, but is a minor effect of in utero exposure to DES. The XY DSD related to insufficient or deficient androgen production or activity during fetal life contributes to the low male sex differentiation of the brain [34]. This will lead to disorders of male gender identity, male behavior and male sexual orientation. Therefore, fetal exposure to any chemicals that can reduce androgen production or action should be considered a transgender risk factor [17]. In 1991, Renish et al. reported that male patients exposed in utero to DES appeared to be feminized [49].

In 2005 and 2020, Kerlin et al. conducted a 5-year qualitative study of DES sons via the Association DES-Sons USA and analyzed a sample of 500 men with confirmed DES exposure. In total, 90 individuals with verified DES exposure identified as transsexual, transgender or gender dysphoric (personal data). In addition, 65 individuals with strongly suspected but not confirmed DES exposure also defined themselves as “transsexual, transgender or gender dysphoric”. Kerlin et al. carried out extensive surveys and interviews with several of them for more than 15 years that enabled a stronger verification of the full scope of DES-related effects (personal data).

Based on a study by Tournaire et al. [21,31], the DES doses administered in France (4050 mg < D < 7300 mg) were lower than those administered in the United States (7550 mg < D < 12,742 mg; doses varied in the different states). These authors investigated DES’s role in clear cell carcinoma development in DES daughters in France and proposed that early fetal exposure (administration beginning during the first trimester of pregnancy which represents most cases) is more important than the dose. We think that this hypothesis is valid also for our DES sons.

Conversely, Titus et al. [50] evaluated a cohort of 2694 DES sons and found little support to the hypothesis that prenatal exposure to DES influences the psychosexual characteristics of adult men. Troisi et al. [51], in a group of 1848 DES sons, reported an odds ratio of 1.4 of having a gay identity or bisexual identity (five of these individuals were transgender women). Similarly, our finding of four transgender women among 253 XY patients who were exposed to DES in utero (1.58%), a much higher rate than in the general population and probably underestimated because no epidemiological investigation has been carried out on this topic, strongly suggests that DES plays a role in male to female transgender development. DES displays antiandrogenic activity in all assays used [52]. Northern blotting and RT-PCR showed a significant downregulation of androgen responsive genes upon exposure to DES [53]. As a xenoestrogen, DES can also stimulate production of sex hormone-binding globulin (SHBG), the major androgen-binding protein in serum that reduces androgen’s ability to enter into the cell [54]. By reducing androgen production, bioactivity and action in target cells, DES must be considered an anti-androgen substance that can alter male sex differentiation of the brain and/or sex differentiation of the internal/external genitalia.

Differences in sex behavior may have correspondence in the brain architecture. Several studies reported an association between brain structure [15,55,56,57] and gender identity. Zhou et al. [58] first showed by brain imaging that the bed nucleus striae terminalis volume differs in men and women, and this is similar in transgender women and women. Garçia-Falgueras et al. [59], using brain imaging, found that the interstitial nucleus of the anterior hypothalamus volume is different between males and females and that its volume in transgender women is within the female range. Luders et al. reported regional gray matter variations in male-to-female transsexualism [55]. Lastly, Mueller et al. analyzed magnetic resonance imaging (MRI) data from transgender men and women and cisgender men and women. Based on their findings, they suggested that transgender persons present a specific brain phenotype [56]. Unfortunately, no MRI data were available for our four patients.

In conclusion, the presented clinical data from our DES cohort, experimental works and the endocrine consequences of environmental disruptors suggest for the first time that male to female transgenderism in DES sons is associated with DES exposure and is not a coincidental finding.

## Figures and Tables

**Table 1:** Clinical features of our four patients with female gender variance, from childhood to adulthood.

Clinical Data	Patient			
	1	2	3	4
(**A**) **During childhood**				
Strong desire to be female	X	X	X	X
Strong preference for wearing female clothes	X	X	X	X
Strong preference for cross-gender role in make-believe play or fantasy play	X	X	X	X
Strong preference for toys, games or activities stereotypically used or engaged in by the female gender	X	0	0	0
Strong preference for playmates of female gender	0	X	X	X
Strong rejection of toys, games and activities typical of the male gender	0	0	0	0
Strong dislike of one’s sexual anatomy	X	X	X	X
Strong desire for the physical sex characteristics that match one’s experienced gender	X	0	0	X
(**B**) **During puberty/adolescence**				
Experiencing the wrong puberty for transgender youth	0	X	X	X
Self-mutilation (external genitalia)	X	0	0	0
Vulnerability	X	X	X	X
Mood disorders	X	X	X	X
Depression/anxiety	0	0	0	X
Maladaptive coping	X	X	X	X
Suicidality	0	0	0	0
Psychiatric hospitalization	0	0	0	0
Institutional discrimination	X	X	0	X
Economic marginalization	X	X	0	X
Social isolation	X	X	0	X
Risk of drug abuse	0	0	0	0
Violence	0	0	0	X
Homelessness	0	0	0	0
(**C**) **In adulthood**				
Gender-affirming surgery	X	X	X	0
Gender-Affirming Hormone Therapy	X	X	X	X

## Data Availability

Data are contained within the article.
